# RACIAL/ETHNIC DIFFERENCES IN COPING RESOURCES AND PURPOSE IN LIFE AMONG OLDER AMERICANS

**DOI:** 10.1093/geroni/igae098.1824

**Published:** 2024-12-31

**Authors:** Man Guo, Yi Wang, Morgan Stangl

**Affiliations:** University of Iowa, Iowa City, Iowa, United States; University of Iowa, Iowa City, Iowa, United States; University of Iowa, Iowa City, Iowa, United States

## Abstract

Purpose in life (PiL), the sense of direction and meaning in life, has been found to be linked to favorable health outcomes in later life, even lower mortality. Less is known about the perception of PiL among racial/ethnic minorities, who often face numerous adversities, but also have strong coping resources derived from religiosity, social support networks, and neighborhood contexts. Using data from the 2016 and 2018 psychosocial assessment of the HRS (N=11,579), this study assessed racial/ethnic differences in PiL among middle-aged and older adults and the role of coping resources (religiosity, social support, neighborhood cohesion) in shaping PiL. ANOVA results showed that compared to White older adults, Black older adults had significantly higher PiL, and Hispanic older adults had significantly lower PiL. Regression results further showed that all the coping resources were positively associated with higher PiL within each racial/ethnic group, with the exception of religiosity for Hispanic older adults. Despite racial/ethnic minority groups’ significantly higher ratings on religiosity and neighborhood cohesion, the positive associations between the three coping resources and PiL were stronger among White older adults than racial/ethnic minority groups. Aside from possible measurement bias or cultural response differences, the findings suggest that racial/ethnic minority older adults may face unique challenges that impact their effective utilization of coping resources, potentially weakening their PiL. Our findings highlight the importance of better understanding factors that promote PiL among racial/ethnic older minorities, especially Hispanics. They also provide insights into culturally tailored approaches to enhance well-being within these communities.

